# Supplementary figures and images for: Prenatal methadone exposure selectively alters protein expression in primary motor cortex: Implications for synaptic function
Source: Front Pharmacol. 2023 Feb 1;14:1124108. doi: 10.3389/fphar.2023.1124108 (PMC9928955; doi:10.3389/fphar.2023.1124108)

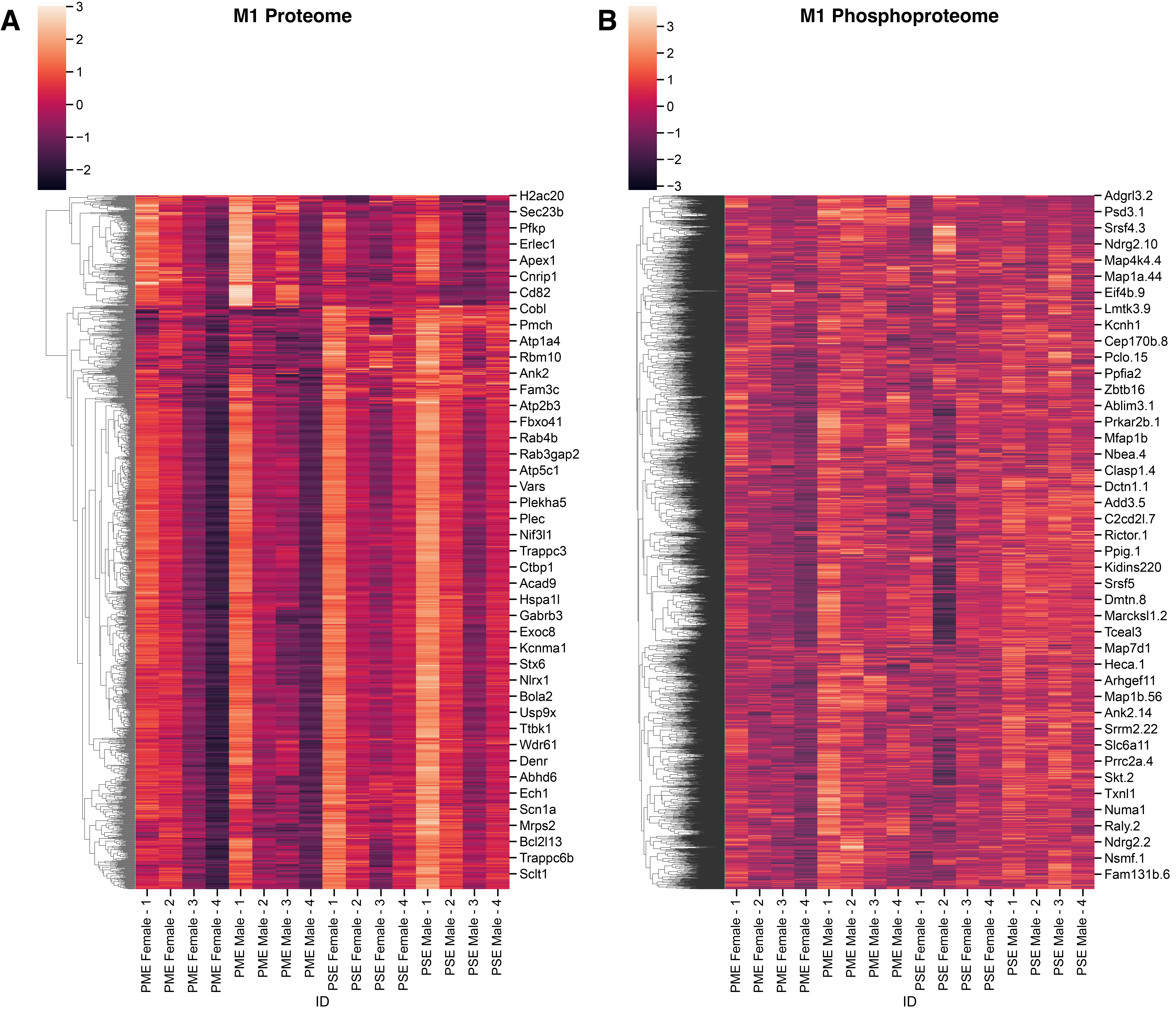

Supplement: Supplementary file 7 [file Image1.jpg]
